# Disassociated relation between plasma tumor necrosis factor-α, interleukin-6 and increased body weight in Amerindian women: A long-term prospective study of natural body weight variation and impaired glucose tolerance

**DOI:** 10.1186/1758-5996-2-38

**Published:** 2010-06-08

**Authors:** Folke Lindgärde, Anders Gottsäter, Bo Ahrén

**Affiliations:** 1 Vascular Center, Lund University, Skåne University Hospital, S-205 02 Malmö, Sweden; 2 Department of Clinical Sciences, Division of Medicine, Lund University, B11 BMC, SE-221 84 Lund, Sweden

## Abstract

**Background:**

Inflammatory cytokines are linked to obesity-related insulin resistance and may predict type 2 diabetes independently of obesity. We previously reported that a majority of a cohort of 73 non-diabetic women with normal plasma (p-)glucose with Amerindian heritage in Lima, Peru, during a 5-year period increased both body weight and p-glucose levels, yet p-insulin was unaltered. A high proportion of palmitoleic acid (16:1n-7) in serum (s) and systolic blood pressure (SBP) were independent predictors of high p-glucose. Whether cytokines also contributed is, however, not known.

**Methods:**

During 5 years we prospectively investigated the relation between changed concentrations of p-tumor necrosis factor (TNF)-α, p-interleukin (IL)-6 and circulating insulin and glucose in relation to the natural variation of body weight. Study variables included anthropometric measurements, p-insulin, TNF-α, IL-6, SBP and the proportion of 16:1n-7 in s-fatty acid composition.

**Results:**

Weight and waist differences correlated negatively to the difference in p-TNF-α but positively to differences in p-IL-6 and p-insulin, whereas the increase of p-glucose from baseline to follow-up did not correlate with changes in levels of the two cytokines. In multiple regression analysis changes of TNF-α and insulin contributed independently to the variance in weight. P-insulin at baseline and weight change were determinants of fasting p-insulin at follow-up. Multiple regression analysis revealed that weight change (t-value = - 2.42; P = 0.018) and waist change (t-value = 2.41; P = 0.019) together with S-16:1n-7 (p < 0.0001) and SBP (p = 0.0005) at baseline were significant predictors of p-glucose at follow-up.

**Conclusion:**

Our prospective study of Amerindian women revealed disassociations between changes in p-TNF-α and p-IL-6 in relation to variation in body weight. A high proportion of s-16:1n-7, SBP at baseline together with weight and waist changes were independent predictors of p-glucose at follow-up. The exact role of the opposite effects and clinical impact of p-TNF-α and p-IL-6 on loss and gain of body weight and indirectly on the development of glucose intolerance is not known.

## Background

Sedentary socioeconomically marginalized women with an Amerindian heritage living in a northern suburb of Lima, the capital of Peru, were in a previous study characterized by normal fasting plasma (p-)glucose levels, but high insulin levels [1,2]. A follow-up after five years revealed that the majority of the women along with increased body weight and fat mass had developed higher fasting p-glucose values possibly concomitant with insufficient insulin secretion. The proportion of palmitoleic acid (16:1n-7) in serum (s) and systolic blood pressure were strong independent determinant*s *of p-glucose concentration 5 years later [3].

In this study, we examined whether cytokines may contribute to this metabolic state as there is a growing body of evidence indicating that obesity may be associated to chronic activation of the innate immune system [4], resulting in progressive impairment of glucose tolerance and type 2 diabetes [5]. However, elevated levels of inflammatory markers may also predict type 2 diabetes independently of obesity [6], and future weight-gain [7]. These results support the view that elevated levels of inflammatory markers occur early in the process, leading to glucose intolerance with or without concomitant weight gain. A possibility is therefore that cytokines, such as interleukin (IL)-6 and tumor necrosis factor (TNF)-α, contribute to the development of insulin resistance and impaired islet function. This is, however, controversial because in a cross-sectional study, neither p-TNF-α nor p-IL-6 levels were independently associated with hepatic or peripheral insulin action [8]. Nevertheless, in people with type 2 diabetes, the circulating p- IL-6 concentration is correlated with adipose tissue mass, rather than with whole-body insulin sensitivity [9], suggesting that IL-6 may be a marker of obesity without any direct contribution to the development of insulin resistance.

Cytokines are, in many ways, involved in the regulations of metabolism and food intake. TNF-α influences energy homeostasis, has an anorexigenic effect on the hypothalamus [10], and a role in the development of neoplastic anorexia [11]. High concentrations of TNF-α and IL-6 are associated with lower muscle strength and endurance [12]. Furthermore, higher levels of TNF-α are associated with greater 5-year decline in thigh muscle area and grip strength [13]. It is obvious that very small alterations in metabolism or food intake can cause a moderate weight decrease.

The present study sought to explore whether a change of circulating TNF-α or IL-6 during follow-up of 5 years in our material of Amerindian women [1-3] was associated with gain or loss of body weight, and whether it predicted fasting insulin and glucose concentrations.

## Methods

### Study participants

The original study [1] consisted of 182 Peruvian Amerindian women living in a northern urban area of Lima. A migration antecedent from indigenous Andean communities in Peru was identified in all participants. The age range was 20-59 with a mean of 41 years. Fatty acid composition in serum was measured in 141 subjects. All of these subjects had normal fasting p-glucose concentrations. This subgroup was invited to participate in a new survey [3] five years later. Seventy-nine subjects attended the second investigation. P-glucose concentrations were available from 73 subjects in both investigations. These 73 women did not differ significantly from the remaining 68 in baseline characteristics of p-concentrations of insulin and glucose, s-palmitoleic acid (16:1n-7) or body weight. None of the participants was taking cholesterol lowering medication, was consuming a special diet, had ongoing cardiovascular disease, or was pregnant.

Approval for the study had been given in 1998 and 1999 by the Ethics Committees of Lund University and San Martin University Hospital in Lima. Furthermore, the follow-up examination was separately approved by the Ethics Committe of San Martin University Hospital. The study was undertaken in accordance with the Helsinki Declaration of 1975, as revised in 1983. Informed consent was obtained from all subjects.

### Study methods

The examinations took place in the morning after an overnight fast at Alternativa, a center for social research and popular education in the district of San Martin, Lima.

### Anthropometric measurements

Trained nurses measured weight, height and waist circumferences. Changes in the respective values were calculated as measurement at follow-up subtracted from the baseline value. Body mass index (BMI; in kg/m^2^) was calculated according to the standard formula. Body fat mass and the percentage fat mass in relation to body mass were determined by measuring the resistance of the body to a low-level electrical current (Biodynamic Model 310e; Biodynamic Research Inc, Seattle, WA). Measurements were performed with subjects lying on a couch for 5 min, and the electrodes were placed on the dorsal surfaces of the right hand and foot.

### Measurement of p-insulin, glucose, TNF- α and IL-6

Serum or plasma was separated from venous blood and stored within 1 hour at -20°C and then brought to Sweden for analysis. P-insulin was measured with double-antibody radioimmunoassay techniques with the use of guinea pig anti-human insulin antibodies and human insulin as the standard (Linco Research, St Charles, MO, USA). P-glucose was measured by using the glucose oxidase procedure. For the indirect determination of insulin sensitivity, the homeostasis model assessment of insulin resistance (HOMA-IR) was calculated as follows [14]: HOMA-IR = [fasting insulin (in pmol/L) x fasting glucose (in mmol/L)/22,5]. P-TNF- α and p-IL-6 were measured by ELISA using commercially available test kits (Pharmingen, San Diego, CA, USA). Detection limits were 0.12 pg/ml and 0.70 pg/ml [15], intraassay coefficients of variation (CV) were 8.8% and 4.4%, and interassay CV were 16.7% and 6.4%, respectively.

### Lipid extraction and serum fatty acid measurements

The fatty acid composition of s-cholesteryl esters was measured as previously described [16]. Serum was extracted with a hexane-isopropanol solution, and cholesteryl esters (only lipid esters that were measured at baseline) were separated from the extract by thin-layer chromatography before interesterification with acidic methanol was performed. Free cholesterol that had been liberated in the reaction was removed by aluminum oxide to avoid contamination of the column. The composition of methylated fatty acids was determined by gas chromatography (25-m NB-351 silica capillary column) with a flame ionization detector and helium as carrier gas. Every 25th sample was a serum control pool. The CV between successive gas chromatography runs was 0.2-5%. The relative amount of fatty acid was expressed as a percentage of the total amount of fatty acids reported.

### Statistical analyses

Data are expressed as mean ± SD. The correlation coefficients between 2 variables were determined by Spearman rank analysis. Differences between measurements at baseline and at follow-up levels were analysed by use of Wilcoxon signed rank tests (Table 1). Because p-concentrations of glucose, TNF-α, IL-6, and insulin values followed a log-normal distribution, logarithmic transformation was used for these variables. Multiple regression analyses were undertaken to examine which variables predicted alterations of weight and waist during the observation period and p-glucose and insulin values at follow-up. The selected variables were forced into the model based on analysis (Table 2) and prior findings such as 16:1-n7 and systolic blood pressure [3]. All statistical analyses were conducted by using the statistical package STATVIEW (version 5.0.1, for Macintosh; SAS Institute Inc, Cary, NC).

**Table 1:** Indices of adiposity and metabolic markers at baseline survey and at follow-up 5 years later.

	*Baseline*	*Follow - up*
N	73	73
Age (y)	40.8 ± 10.8	
Body weight (kg)	60.0 ± 13.9	63.1 ± 15.0***
Waist (cm)	84.1 ± 12.5	91.3***
BMI (kg/m^2^)	25.9 ± 5.5	27.2***
Percentage fat mass (%)	35.2 ± 5.4	36.7 ± 5.1**
Fat mass kg	23.1 ± 8.1	23.7 ± 8.0**
P-glucose (mmol/L)	4.12 ± 0.45	5.01 ± 1.25***
Insulin (pmol/L)	80.9 ± 42.6	83.4 ± 62.9
HOMA -IR	15.2 ± 9.1	19.0 ± 15.5**
Interleukin-6 (pg/mL)	2.03 ± 2.49	1.89 ± 1.50
TNF-α(pg/mL)	4.43 ± 5.98	3.35 ± 3.85**

All values are means ± SD. HOMA-IR = homeostasis model of assessment ratio. TNF-α = tumor necrosis factor alpha. Significant differences during the follow-up period are represented by < 0.05*, < 0.01** and < 0.001*** (Wilcoxon signed rank test).

**Table 2:** Spearman rank correlation between differences (diff) between baseline and follow-up values. R- and (P-values).

	Weight diff	Waist diff	TNF-α diff	IL-6 diff	Insulin diff	Glucose diff
Weight diff	-	<.0001	-.35 (.0031)	.25 (.0312)	.27 (.0194)	NS
Waist diff		-	-. 26 (.025)	.25 (.0298)	.25 (.0298)	NS
TNF-α diff			-	NS	-.23 (.045)	NS
IL-6 diff				-	NS	NS
Insulin diff					-	-.27 (.018)
Glucose diff						-

TNF-α = tumor necrosis factor alpha. IL-6 = interleukin-6

## Results

### Body composition

As can be seen in Table 1 body weight, BMI, waist circumference, fat mass and fat as a percentage of body weight increased significantly. Twenty-one of the women lost body weight (-2.0 ± 1.8 kg) while 52 gained body weight (+5.6 ± 4.0 kg).

### Glucose and insulin

Mean p-glucose increased from 4.12 ± 0.45 to 5.01 ± 1.25 mmol/l (p < 0.001). In contrast p-insulin was unchanged during the 5 years. Consequently, when calculating insulin resistance according the homeostasis model of assessment ratio, HOMA-IR values increased significantly.

### TNF-α and IL-6

The decrease in p-IL-6 from 2.03 ± 2.49 to 1.89 ± 1.50 pg/ml did not reach significance while the decrease in TNF-α from 4.43 ± 1.75 to 3.35 ± 3.85 was significant (p = 0.003). In subjects who lost body weight p-TNF-α and p-IL-6 concentrations were unchanged during the follow-up period. In women who gained body weight. p-TNF-α concentration decreased from 4.9 ± 6.7 pg/ml to 3.3 ± 3.9 (p < 0.0001) whereas p-IL-6 increased from 1.75 ± 2.27 to 2.03 ± 1.63 (p = 0.002).

### Weight change in relation to differences in waist circumference, p-cytokine, insulin and glucose concentrations between baseline and follow-up examinations

Differences were calculated as the value at follow-up subtracted from the value at baseline. Spearman rank correlation was used to analyse the associations (Table 2). Weight and waist differences correlated negatively to the difference in p-TNF-α but positively to differences in p-IL-6 and p-insulin, whereas the increase of p-glucose from baseline to follow-up did not correlate with changes in levels of the two cytokines. The decrease of p-TNF-α during the observation period correlated negatively to changes of p-insulin.

### Predictors of weight and waist increase

Multiple regression analysis was conducted to sort out independent contributions of differences in p-IL-6, TNF-α and insulin during the observation period to the variance in weight and waist circumference. Both changes of TNF-α and insulin contributed independently to the variance in weight (Table 3) and together with p-IL-6 in waist circumference (Table 4).

**Table 3:** Multiple regression with weight, insulin, p-TNF-α and p-IL-6 variables from baseline and differences of TNF-α, IL-6 and insulin during 5 years as independent variables and body weight change during 5 years as dependent variable.

	Coefficient	SE	P-value	Standardized coefficient
Intercept	0.03	2.40	0.99	0.03
P-TNF-α difference	-0.50	0.21	0.020	-0.40
P-IL-6 difference	0.70	0.36	0.054	0.39
P-insulin difference	0.04	0.01	0.0003	0.41
Weight baseline	0.004	0.04	0.909	0.01
P-insulin baseline	0.03	0.02	0.024	0.27
P-TNF-α baseline	-0.17	0.14	0.235	-0.20
P-IL-6 baseline	0.38	0.39	0.338	0.19

**Table 4:** Multiple regression with waist, insulin, p-TNF-α and p-IL-6 variables from baseline and changes of p-TNF- α, IL-6 and insulin during 5 years as independent variables and waist circumference change during 5 years as dependent variable.

	Coefficient	SE	P-value	Standardized coefficient
Intercept	-0.93	5.63	0.87	-0.93
P-TNF-α difference	-0.59	0.24	0.016	-0.43
P-IL-6 difference	1.14	0.41	0.007	0.60
P-insulin difference	0.02	0.01	0.025	0.26
Waist baseline	-0.05	0.05	0.15	0.18
P-insulin baseline	0.02	0.02	0.021	0.29
P-TNF-α baseline	-0.16	0.16	0.316	-0.18
P-IL-6 baseline	0.71	0.45	0.117	0.34

### Predictors of plasma insulin and glucose values at follow-up

The multiple regression analysis revealed that weight change and p-insulin at baseline were the only significant predictors of fasting p-insulin at follow-up (Table 5). Neither p-TNF-α nor p-IL-6 and age at baseline, or the variations of the two cytokines during the observation period contributed independently to the variance of p-insulin at follow-up.

**Table 5:** Multiple regression with p-TNF-α, IL-6 and insulin at baseline together with changes of weight, waist, p-TNF-α, IL-6 and insulin as independent variables and p-insulin at follow-up as dependent variable.

	Coefficient	SE	P-value	Standardized coefficient
Intercept	0.71	0.22	0.002	0.71
P-TNF-α difference	-0.06	0.09	0.55	-0.07
P-IL-6 difference	0.11	0.07	0.10	0.22
Weight difference	0.02	0.01	0.0006	0.53
Waist difference	-0.01	0.01	0.13	-0.21
P-insulin baseline	0.62	0.11	< 0.0001	0.55
P-TNF-α baseline	0.04	0.07	0.60	0.06
P-IL-6 baseline	-0.03	0.07	0.72	-0.05
Age	-0.01	0.01	0.84	-0.02

In order to sort out whether weight and waist changes together with baseline measurements were associated with p-glucose at follow-up, a partial correlation matrix was studied. The analysis gave the following R values with p-glucose at follow-up; weight change = - 0.38, waist change = 0.35, S-16:1n-7 = 0.57, systolic blood pressure = - 0.38, p-insulin = 0.19, p-TNF-α = -0.04 and p-IL-6 = 0.34.

In a multiple regression analysis including these variables and age as independent determinants both anthropometric measurements together with S-16:1n-7, SBP and p-glucose at baseline were significant determinants and explained 49.0% of the variability (Table 6).When b-glucose was omitted from this analysis, 45.4% of the variability was explained by the other factors. P-concentrations of insulin and IL-6 and age did not contribute independently to the variability of p-glucose measured five years later.

**Table 6:** Multiple regression with changes of weight and waist together with baseline values of the proportion of palmitoleic acid (16:1n-7) in serum, systolic blood pressure (SBP), plasma (p-) concentrations of glucose, IL-6 and insulin as independent variables and p-glucose at follow-up as dependent variable.

	Coefficient	SE	P-value	Standardized coefficient
Intercept	0.99	0.22	< 0.001	0.99
Weight change	- 0.01	0.002	0.007	-0.43
Waist change	0.01	0.002	0.0125	0.38
S-16:l-n7 baseline	0.34	0.07	< 0.0001	0.54
SBP baseline	-0.43	0.19	0.0005	-0.41
P-glucose baseline	0.37	0.18	0.044	0.23
P-insulin baseline	0.07	0.04	0.086	0.19
P-IL-6 baseline	0.02	0.02	0.175	0.13
Age	0.01	0.001	0.74	-0.04

## Discussion

Epidemiological studies have demonstrated that deteriorating glucose tolerance is closely associated with increasing body fat mass and obesity especially in individuals with an Amerindian heritage [17,18]. In the present prospective study of 73 Peruvian Amerindian women with fasting p-glucose within the normal range, the proportion of palmitoleic acid (16:1n-7) in serum and systolic blood pressure were strong independent determinants of higher p-glucose values 5 years after the baseline examination, and this was seen in association with unaltered p-insulin [3]. This suggests that insulin resistance with adequate islet compensation existed at the time of the first examination [1], whereas after the 5 year period, insulin secretion from β-cells was insufficient to cope with a further deterioration of peripheral insulin sensitivity.

Twenty-one of the women lost body weight (-2.0 ± 1.8 kg) while 52 gained body weight (+5.6 ± 4.0 kg). P-TNF-α and p-IL-6 concentrations were unchanged in women who lost weight whereas p-TNF-α concentration decreased (p < 0.0001) and p-IL-6 values increased (P = 0.0002) in women who gained weight during the observation period. We now report that differences in body weights and waist circumferences during the observation period correlated negatively to changes in p-TNF-α and positively with p-IL-6 values, (Table 2), and that measured differences in p-TNF-α contributed independently to the variance in weight (Table 3) and together with changed p-IL-6 concentrations were determinants of the variation of waist circumferences that took place during the 5 years study period (Table 4). This disassociation between differences of TNF-α and p-IL-6 in samples from the baseline and follow-up surveys in relation to natural variation of body weight and waist is a novel finding. The alteration of body weight, in contrast to change in waist circumference, together with p-insulin value at baseline were strong predictors of fasting insulin concentration at the follow-up survey (Table 5). Neither p-TNF-α nor p-IL-6 at baseline or the variation of the two cytokines during the observation period, on the other hand, contributed independently to the variance of p-insulin at follow-up.

In our previous report [3] serum 16:1n-7 was closely related to a cluster of risk factors associated with the metabolic syndrome and an increased risk of glucose intolerance and cardiovascular diseases. A majority of the participants had higher fasting p-glucose concentrations at follow-up (86%), but only two had developed diabetes. This increased glycaemia may represent the first step towards manifest diabetes as a consequence of primarily β-cell dysfunction with or without simultaneous increasing insulin resistance [19]. P-glucose concentration in the population increased during the observation period to the same degree independently of whether women gained lost body weight. A multiple regression analysis with p-glucose concentration at follow-up as dependent variable revealed disassociation between changes of body weight and waist in relation to p-glucose concentration at follow-up (Table 6).

These findings suggest a contributing role for the two cytokines indirectly to changes in glucose metabolism through their diametrically opposed influences on weight and waist.The findings of the present study indicate that the increase in p-glucose values during the observation period first of all was related to fatty acid metabolism. In human obesity 16:1n-7 in serum cholesterol ester strongly correlates with indexes of adiposity [20]. Also in the present study, s-16:1n-7 proportion was related to anthropometric measurements at baseline, such as waist circumference (P = 0.006), and percentage body fat (P = 0.0003), but not to p-TNF-α and p-IL-6 or to changes in weight, waist circumference or in levels of the two cytokines. Thus, in addition to the close association between s-16:1n-7 and deterioration of fasting glucose concentration, changes in body weight and waist circumference were also determinants of p-glucose at follow-up.

The intimate relationship between visceral obesity and inflammation was underscored by the findings that intentional weight reduction causes down regulation of inflammation in visceral fat as well in the circulation [21-23]. This was in the present study illustrated by the fact that p-TNF-α and IL-6 concentrations were unchanged in subjects who lost body weight. However, at the same time waist circumferences increased significantly, whereas the percentage of body fat was unchanged (data not shown). Taken together, these findings indicate that lean body mass has to some extent decreased and subcutaneous body fat has been redistributed.

Our study population is characterized by a sedentary lifestyle and low cardiorespiratory fitness [2]. It is most likely that the increases of body weight and waist circumference during the 5 years were due to reduced energy expenditure. The mechanism of energy expenditure involves inflammatory cytokines, such as TNF-α, IL-1, and IL-6, levels of which are positively associated with energy expenditure [24]. In transgenic mice with deficiencies in these cytokines or their receptors, body weight gain is enhanced [25]. In contrast, when cytokine activity is stimulated, energy expenditure is increased, and body weight gain is attenuated [26,27]. Thus, the reduction of p-TNF-α and the concomitant weight gain in our study group is in line with these observations. Inflammation may have two different roles in the regulation of metabolism. The first may be inhibition of insulin sensitivity (a negative effect), as suggested by many studies of low grade inflammation [28-30], and the second may be induction of energy expenditure (a positive effect) [27,31].

A strength of our study is that we followed a homogenous group of women with normal p-glucose values and hyperinsulinaemia [1] for a relatively long period of time, 5 years [3]. Another strength of the study is that no active intervention (diet or exercise programmes) took place since all women had normal fasting p-glucose concentration at the baseline survey. Accordingly it was possible to examine relationships between circulating TNF-α, IL-6, insulin and glucose taking the natural variation of body weight into account.

It is well known that advancing age plays an important role in the progressive β-cell failure that characterises type 2 diabetes. However, age did not correlate with any measured variable at baseline and was not a determinant of p-insulin (table 5) or p-glucose (table 6) at follow-up. These observations indicate that age in the present study may not explain the increase of insulin resistance (table 1) during the 5-year observation period.

The report by Chan et al [32] is in agreement with our observations They found that obesity, dyslipidemia, IL-6, and TNF-α were the principal explanatory variables for the various components of the metabolic syndrome in Caucasian non-diabetic subjects, with IL-6 and TNF-α having different explanatory variables and effects [32]. Our findings of disassociations between p-TNF-α and p-IL-6 in relation to changes of body weight and waist circumference are thus partly consistent with previous work and would suggest an importance for these cytokines also in this population of Amerindian women.

## Conclusions

The present prospective study revealed disassociation between the changes in p-TNF-α and p-IL-6 concentrations in samples from the baseline survey and the follow-up measurements in relation to body weight at follow-up five years later when natural variation of body weight was taken into account. The exact role of the opposite effects and clinical impact of p-TNF-α and p-IL-6 on development of insulin resistance and perhaps type 2 diabetes in subjects with unintentional wasting and women who gained body weight now needs to be examined further.

## Competing interests

The authors declare that they have no competing interests.

## Authors' contributions

FL and BA were responsible for the study design. FL was responsible for data collection, BA and AG were responsible for data analysis, and all authors participated in writing the manuscript.
